# Soft tissue perineurioma involving the kidney: a report of two cases with an emphasis on differential diagnosis

**DOI:** 10.1186/s13000-021-01149-5

**Published:** 2021-09-30

**Authors:** Tian-shi Ma, Ling Zhou, Quan Zhou, Xiang-lei He, Ming Zhao

**Affiliations:** 1 Department of Pathology, Laboratory Medicine Center, Zhejiang Provincial People’s Hospital, People’s Hospital of Hangzhou Medical College, 310014 Hangzhou, Zhejiang China; 2 Department of Pathology, People’s Hospital of Lichuan, 445499 Lichuan, Hubei China; 3 Department of Pathology, Jiaxing Second Hospital, 314099 Jiaxing, Zhejiang China; 4 Department of Pathology, Zhejiang Provincial People’s Hospital, People’s Hospital of Hangzhou Medical College, 158 Shangtang Road, 310014 Hangzhou, Zhejiang China

**Keywords:** Soft tissue perineurioma, kidney, molecular genetics, dedifferentiated liposarcoma, low-grade fibromyxoid sarcoma

## Abstract

**Background:**

Soft tissue perineurioma of the kidney is rare, with only a few reported cases. We report two additional cases with histologic, immunohistochemical and genetic analyses.

**Case presentation:**

Both tumors were from adults (1 female aged 49 years and 1 male aged 42 years) and grossly had maximum diameters of 6.5 and 10 cm, respectively. The tumors were overall well circumscribed but unencapsulated, with focally entrapped benign native renal tubules in one case; both tumors seemed to arise in the capsular areas. The tumors had histologic and immunohistochemical profiles consistent with soft tissue perineurioma. Fluorescence in situ hybridization analyses demonstrated that the tumors were negative for amplification of *MDM2* and rearrangements of *ESWR1, FUS*, and *KMT2A*. Targeted next-generation sequencing revealed a low tumor mutation burden and likely pathogenic mutations (*CYP2B6* and *FLT1* mutations for 1 each). Follow-up data were available for both patients; neither had tumor recurrence or metastasis.

**Conclusions:**

In conclusion, renal perineurioma is rare, usually arises in the capsular areas, and is cured by resection. Low-grade dedifferentiated liposarcoma and low-grade fibromyxoid sarcoma as well as other spindle cell lesions should be considered in the differential diagnosis.

## Background

Perineurioma is a rare and usually benign peripheral nerve sheath tumor composed entirely of cells resembling normal perineurium that includes soft tissue, intraneural, sclerosing and reticular variants [1, 2]. Soft tissue perineurioma is morphologically characterized by slender spindle cells with delicate bipolar cytoplasm and wavy or tapering nuclei arranged in predominantly storiform or whorled growth patterns [2]. In immunohistochemistry (IHC) evaluations, perineurioma consistently expresses epithelial membrane antigen (EMA). Claudin-1 and glucose transporter 1 (GLUT1) are also often positive, and CD34 is expressed in approximately 60 % of cases, while staining for S100 protein, SOX10, and glial fibrillary acidic protein (GFAP) is negative[2–4]. Genetically, soft tissue perineurioma is characterized by deletions of 22q12 and mutations in *NF2* or deletions of 17q11 (including *NF1*)[5].

Soft tissue perineurioma mostly affects the superficial soft tissues of the extremities and trunk, with approximately 30 % developing in deep soft tissue and very rarely in visceral locations [2]. Soft tissue perineurioma involving the kidney is exceptional, and to our knowledge, only sporadic cases have been reported in the English literature thus far [6–10]. In this article, we present 2 additional cases of soft tissue perineurioma of the kidney and discuss the differential diagnosis.

## Case presentation

### Case1

**Fig. 1:**
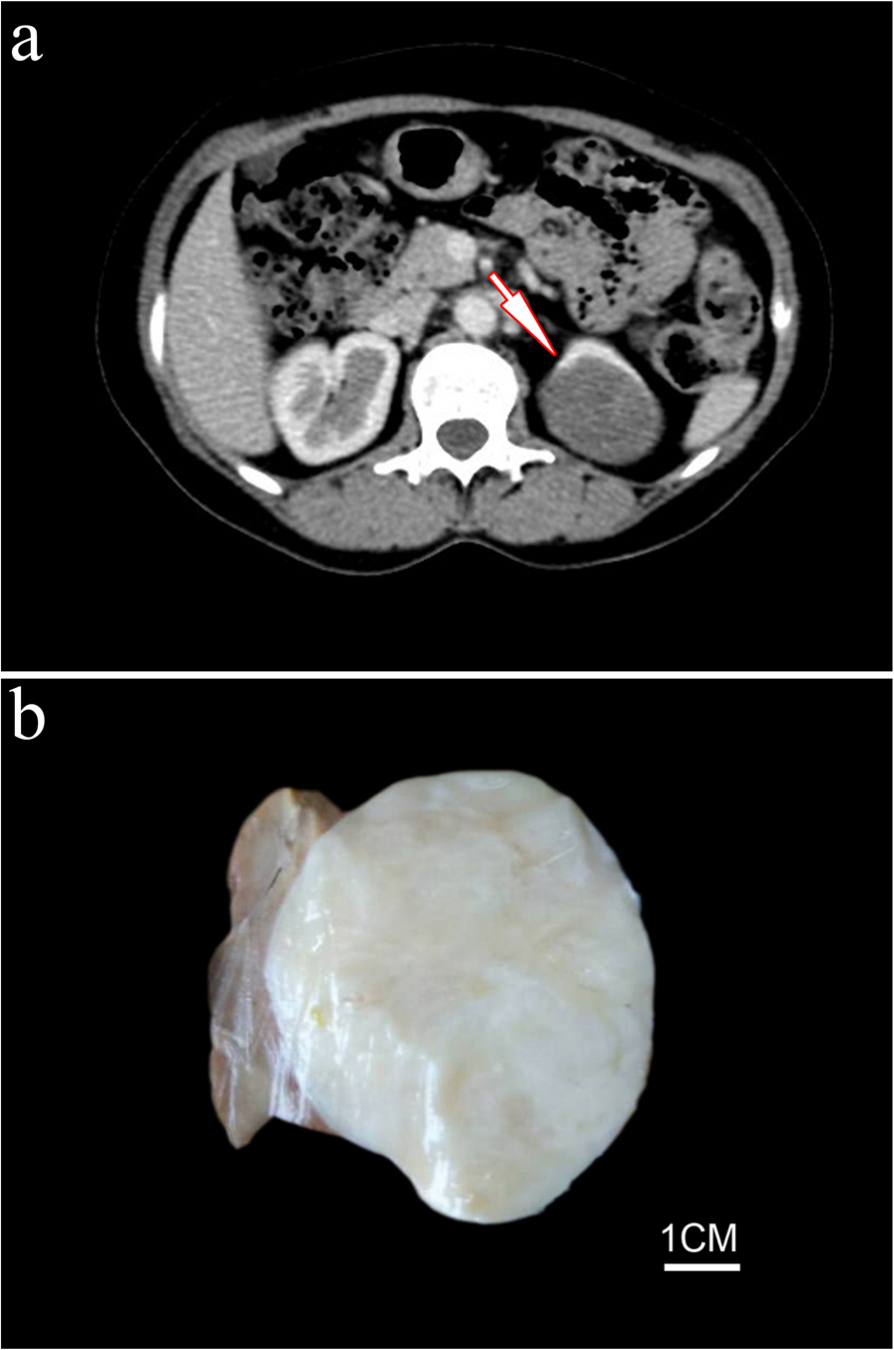
Case 1. CT scan showed a sharply defined mass involving the upper pole of the left kidney (**A**, *as indicated by arrow*). Gross examination revealed a well-demarcated, round and whitish, solid tumor (**B**)

A previously healthy 49-year-old female patient was admitted to the hospital due to incidental discovery of a solid mass in the left kidney by abdominal ultrasound. A computed tomography (CT) scan showed a sharply defined, heterogeneously enhancing mass involving the upper pole of the left kidney measuring 5.7 cm×4.0 cm in size (Fig. 1A). With the suspicion of a malignant lesion, the patient underwent laparoscopic left partial nephrectomy (including tumor and the surrounding renal parenchyma). The patient recovered well after the operation and had no evidence of tumor recurrence or metastasis at 66 months of follow-up.

**Fig. 2:**
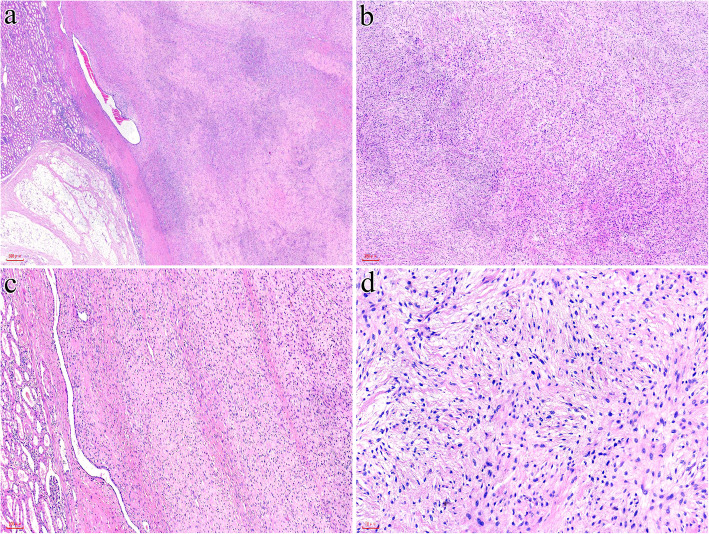
Microscopically, the tumor was unencapsulated with focally entrapped benign renal tubules (**A**). The tumor cells were arranged in interwoven fascicles or storiform patterns in a myxoid to collagenous matrix (**B, C**). The tumor cells were elongated, spindle-shaped cells with ovoid to tapering, bland-appearing nuclei (**D**)

Gross examination of the resection specimen revealed a well-demarcated, round and whitish tumor with solid and firm texture (6.5 cm×5.0 cm×4.0 cm) (Fig. 1B). Microscopically, the tumor was well circumscribed overall but unencapsulated, with focally entrapped benign dilated native renal tubules (Fig. 2A).The tumor cells were arranged in concentric whorls, in interwoven fascicles or in a storiform pattern, set in varying amounts of myxoid and collagenous matrix (Fig. 2B, C). The tumor cells were elongated, spindle-shaped cells with minimal, slightly eosinophilic cytoplasm and ovoid to tapering, bland-appearing nuclei (Fig. 2D). No mitosis or tumor necrosis were identified. Vascularities were scarce and stag-horn shaped, and dilated vessels were occasionally seen.

**Fig. 3:**
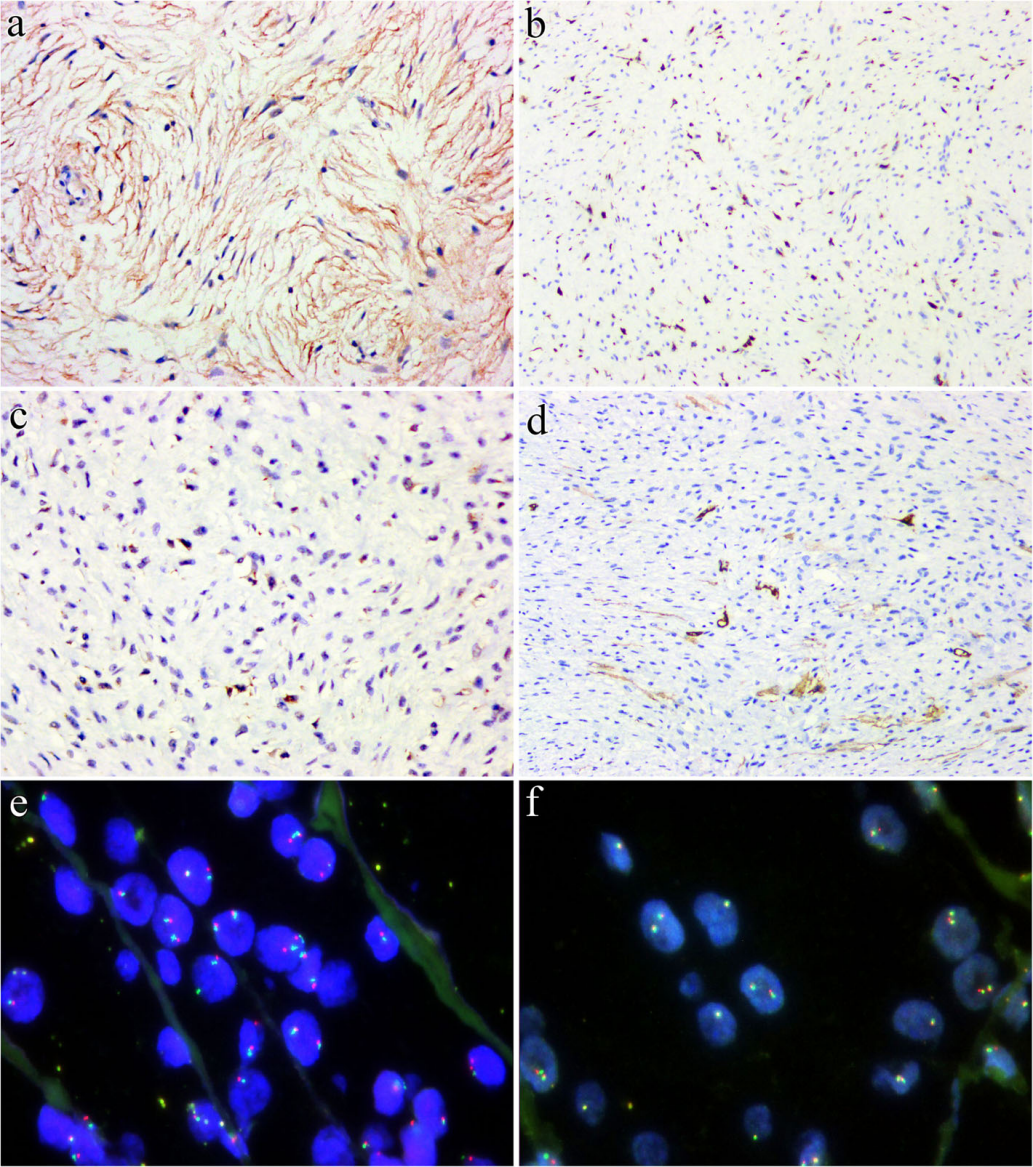
Immunohistochemically, the tumor cells showed diffuse and faint EMA positivity (**A**) and focally positive staining for MUC4 (**B**), claudin-1 (**C**), and CD34 (**D**). FISH analysis revealed that the tumor cells were negative for *MDM2* amplification (**E**, *red signals: MDM2; green signals: CEP12*) and *FUS* rearrangement (**F**, *red signals: 5’ FUS; green signals: 3’ FUS*)

**Fig. 4:**
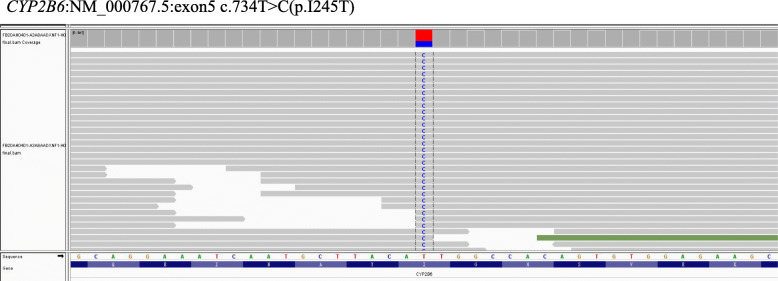
Integrative Genomics Viewer (IGV) split-screen view of read alignments of the identified mutations of *CYP2B6*:NM_000767.5:*exon5 c.734T > C(p.I245T)* in case 1

IHC revealed that the tumor cells showed diffuse and faint EMA positivity with a delicate bipolar staining pattern (Fig. 3A), focally positive staining for MUC4 (in less than 10 % tumor cells)(Fig. 3B), claudin-1(in 10 %-15 % tumor cells) (Fig. 3C) and CD34 (in less than 5 % tumor cells with a similar staining pattern with EMA)(Fig. 3D), and negative staining for GLUT1, S100 protein, estrogen receptor, progesterone receptor, DOG1, smooth muscle actin (SMA), HMB45 and STAT6. Fluorescence in situ hybridization (FISH) analyses demonstrated that the tumor cells were negative for amplification of *MDM2* (Fig. 3E), and rearrangements of *FUS* (Fig. 3F), *EWSR1,* and *KMT2A*. Genetic testing using targeted next-generation sequencing (NGS) for 425 cancer-relevant genes (Gene seqPrime) revealed a low tumor mutation burden (TMB) (1.1 mutations per megabase) and a likely pathogenic mutation of *CYP2B6 (NM_000767.5)* at chromosome 19:41,515,212 of *exon5 c.734T > C(p.I245T*, missense variant), which has a variant allele frequency (VAF) of 25.0 % (Fig. 4). No genomic alterations in *NF1* or *NF2* were present.

### Case 2

A 42-year-old man presented with abdominal discomfort of 1 month. The physical examination revealed a hard mass over his left upper quadrant and knocking tenderness in the left flank area. His medical history was unremarkable, and laboratory findings revealed no abnormalities. An abdominal CT scan indicated a large tumor with a hypodense lesion and a well-defined margin in the left perirenal space. A left partial nephrectomy was performed. The patient recovered well after surgery, and no evidence of local recurrence or distant metastasis was noted on imaging 24 months later.

**Fig. 5:**
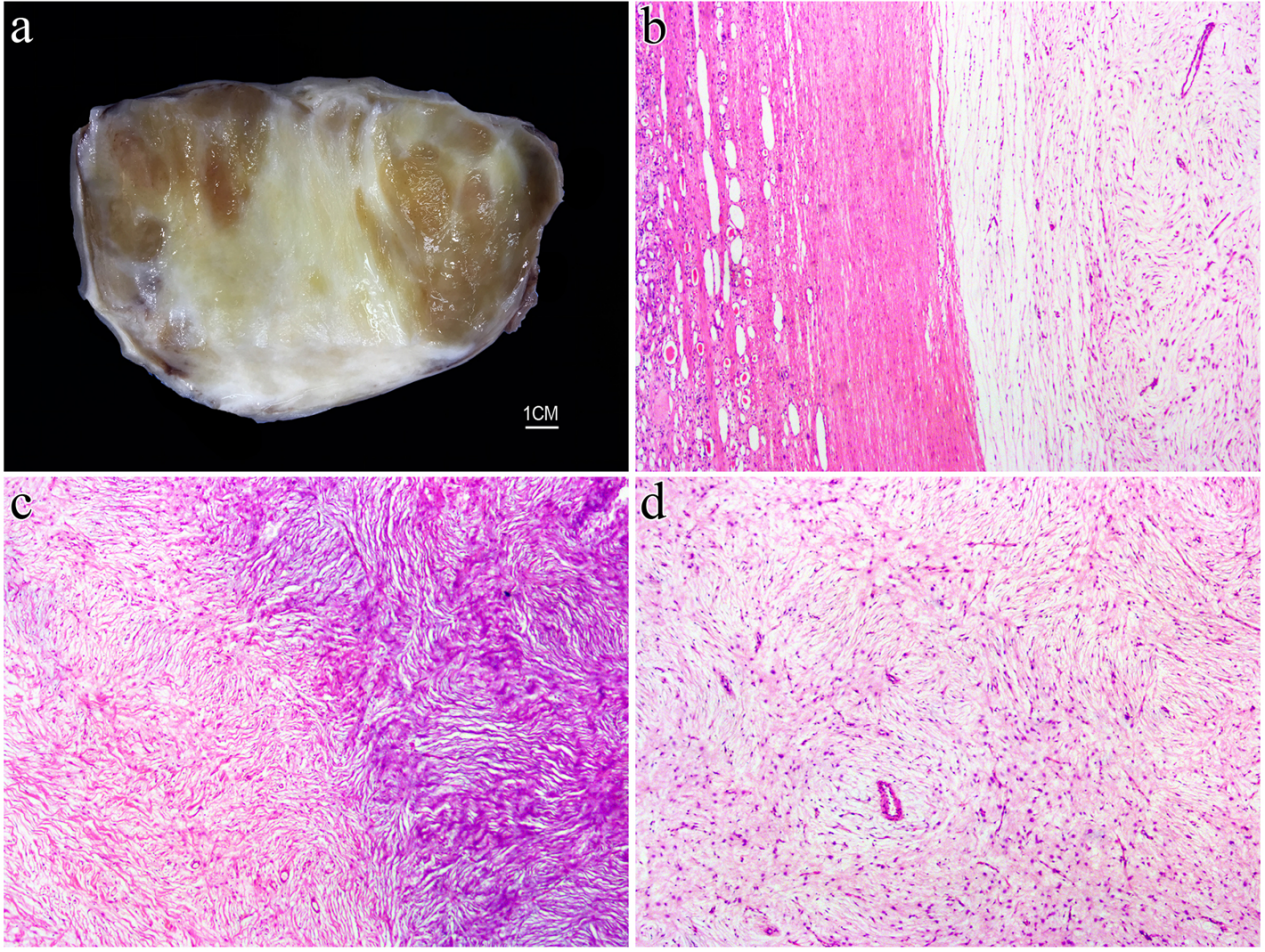
Case 2. Macroscopic examination showed a well-defined tumor with a clear boundary to the surrounding thin renal tissue that was white to yellowish in color (**A**). The tumor was clearly demarcated from the surrounding renal parenchyma and seemed to originate from the renal capsule (**B**). The tumor showed alternating zones of collagenous and mucinous stroma (**C**) and consisted of spindle cells with elongated bipolar cytoplasmic processes arranged in storiform and whorled patterns (**D**)

Grossly, the tumor measured 10 cm in maximum diameter and was well defined with a clear boundary separating the tumor from the surrounding thin renal tissue. The tumor was white to yellowish in color and soft to firm in consistency (Fig. 5A). Histologically, at lower magnification, the tumor was clearly demarcated from the surrounding renal parenchyma, which seemed to originate from the renal capsule (Fig. 5B). In general, the tumor was hypocellular and showed alternating zones of collagenous and mucinous stroma (Fig. 5C). It was composed of spindle cells with tapering nuclei and elongated bipolar cytoplasmic processes arranged in a storiform and whorled architecture surrounding thin-walled small blood vessels (Fig. 5D). No atypia, mitosis or tumor necrosis was noted.

**Fig. 6:**
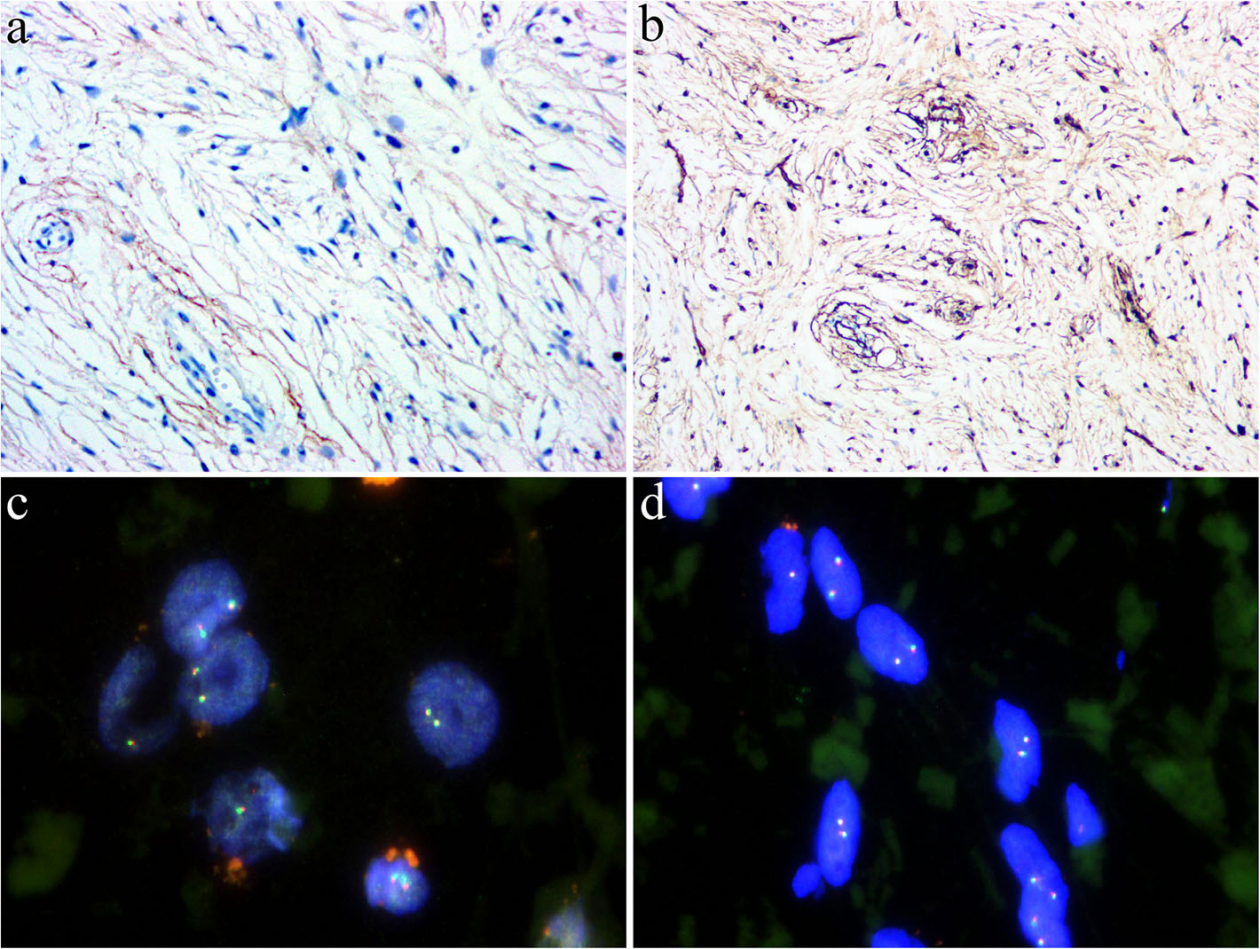
The tumor cells exhibited diffuse and weak expression of EMA (**A**) and CD34 (**B**). In FISH analyses, the tumor cells were negative for rearrangements of *EWSR1* (**C**, *red signals: 5’ of EWSR1; green signals: 3’ of EWSR1*) and *KMT2A* (**D**, *red signals: 5’ of KMT2A; green signals: 3’ of KMT2A*)

**Fig. 7:**
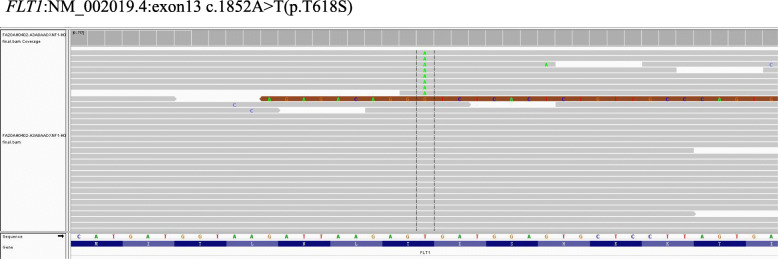
Integrative Genomics Viewer (IGV) split-screen view of read alignments of the identified mutations of *FLT1*:NM_002019.4:*exon13 c.1852 A > T(p.T618S)* in case 2

IHC analyses revealed that the tumor cells were positive for EMA (Fig. 6A) and CD34 (Fig. 6B) and negative for claudin-1, GLUT1, S100 protein, MUC4, STAT6, SMA, HMB45 and P16. FISH studies demonstrated that the tumor was negative for amplification of *MDM2* and rearrangements of *EWSR1* (Fig. 6C), *FUS,* and *KMT2A* (Fig. 6D). Genetic testing using targeted NGS for 425 cancer-relevant genes (Gene seqPrime) revealed a low level of TMB (0 mutations per megabase) and a likely pathogenic mutation of *FLT1 (NM_002019.4)* at chromosome 13:28,964,050 with *exon 13 c.1852 A > T(p.T618S*, missense variant), which has a VAF of 1.5 % **(**Fig. 7). No genomic alterations in *NF1* or *NF2* were present.

## Discussion and conclusions

Soft tissue perineurioma involving the retroperitoneum is rare, and that arising in the kidney is even rarer. In Hornick and Fletcher’s [2] report, only 3/81 (3.7 %) of soft tissue perineuriomas originated in the retroperitoneum, and none was specified to affect the kidney.

A MEDLINE search revealed 6 cases (in 4 patients) of renal perineurioma in English-language publications[6–10](References[7]and[8] reported the same case). At present, we present two additional cases of soft tissue perineurioma involving the kidney, both of which seemed to arise in the capsular areas of the kidney. A summary of the 8 cases of renal perineurioma reported in the literature, including the 2 cases reported in this article, found that these tumors mainly occurred in adults and rarely in children, and all were sporadically without neurofibromatosis type 1 or 2, and they could be discovered accidentally or presented with symptoms due to local compression and irritation (Table 1). Compared with its superficial counterparts, renal perineurioma has a relatively larger diameter, as evidenced by the findings of a previous study that deep-seated tumors were larger in size than subcutaneous tumors[2]. Soft tissue perineurioma is typically benign and rarely recurs. Case 1 in our study showed entrapment of benign native renal tubules, which suggested locally invasive growth and gave an impression of a low-grade malignant tumor. However, atypical histologic features, including scattered pleomorphic tumor cells and infiltrative margins, usually have no clinical significance in the absence of frankly malignant features[2]. Including our 2 cases, all the reported patients with renal perineurioma underwent surgery alone, and none had tumor recurrences during follow-up when available [6–10].

**Table.1:** Summary of the clinicopathologic features of soft tissue perineurioma of the kidney

Study	Case No.	Sex/Age	Clinical manifestations	Lateral/Size	Follow-up(m)
Kahn et al. [6]	1	Female/66 years	Incidentally identified during evaluation of hypertension and proteinuria	NA	NA
Val-Bernal et al. [7] ^a^ and García-Valtuille et al. [8] ^a^	2	Female/7 years	Fever (38.5 °C), nausea and abdominal pain.	Right/3 cm	NA
Huang et al. [9]	3^b^	Male/25years	Progressive left flank distention and dyspepsia for 3 months	Left/14 cm	NED/24
	4^b^			Left/10 cm	
	5^b^			Right/6 cm	
Gan et al. [10]	6	Male/40years	Incidentally discovered in the nonfunctioning graft by routine imaging surveillance	Right transplanted kidney/12 cm	NA
Current case 1	7	Female/49years	Incidentally discovered by abdominal ultrasound	Left/6.5 cm	NED/66
Current case 2	8	Male/42years	Abdominal discomfort of 1 month’s duration	Left/12 cm	NED/24

*NED*: no evidence of disease

^a^References [7] and [8] reported the same case.

^b^Cases 3–5 were concurrent in the same patient.

With regard to soft tissue perineurioma of the kidney, the differential diagnoses were broad and principally included neurofibroma, schwannoma, solitary fibrous tumor, and spindle cell predominant angiomyolipoma in the kidney. Familiarity with its unique morphological features, including storiform or whorled arrangements of slender spindle cells with delicate bipolar cytoplasmic processes with wavy or tapering nuclei, supplemented by the frequent immunoexpression of EMA, claudin-1 and GLUT1 as well as absence of expression of S100 protein, SOX10, STAT6, HMB45, and SMA/desmin, can usually distinguish renal perineurioma from the other entities mentioned above.

Clinically, the most important differential diagnosis is from low-grade dedifferentiated liposarcoma (DDLPS) and low-grade fibromyxoid sarcoma (LGFMS), both of which can extend into and become secondarily involved in the kidney as a primary tumor in the retroperitoneal regions. DDLPS by definition is an atypical lipomatous tumor/well-differentiated liposarcoma showing transition to nonlipogenic sarcoma, which in most cases is of high grade and uncommonly exhibits low-grade histologic features [11]. Low-grade DDLPS is characterized by the presence of relatively uniform fibroblastic-like spindle cells with moderate cellularity and mild nuclear atypia, often rearranged in fascicular and infrequently storiform patterns, which may mimic soft tissue perineurioma histologically. In addition, CD34 has also been expressed in a subset of DDLPS [12], and rare soft tissue perineuriomas may show “pseudolipoblastic” morphology [13], further complicating the differential diagnosis. Perhaps the most difficult situation is to distinguish between soft tissue perineurioma of the kidney with DDLPS with a peculiar meningothelial-like whorl growth pattern [14, 15], especially on biopsy specimens. This distinctive variant of DDLPS is usually located in the retroperitoneum and shares many features with soft tissue perineurioma, including low-grade spindle cells with concentric distributions and frequent expression of claudin-1 by IHC [14]. However, DDLPS with meningothelial-like whorls is commonly associated with metaplastic bone formation and exhibits coexpression of P16, CDK4, and MDM2 [14], markers that are usually negative in soft tissue perineurioma. In difficult cases, FISH analysis for detecting 12q14-15 (including *MDM2* and *CDK4*) amplification, the genetic hallmark of DDLPS, can serve as a robust tool to distinguish DDLPS from soft tissue perineurioma.

Perhaps the closest histologic mimic of soft tissue perineurioma of the kidney is LGFMS, which arises most commonly in deep soft tissue of the extremities and occasionally in the abdominal cavity and retroperitoneum [16]. LGFMS is a low-grade malignant fibroblastic tumor and is related to sclerosing epithelioid fibrosarcoma (SEF) both morphologically and molecularly. LGFMS demonstrates considerable histologic and immunohistochemical overlaps with soft tissue perineurioma, including deceptive bland spindle cells with fascicular or whoring patterns in alternating collagenous and myxoid stroma. By IHC both tumors largely express EMA. In addition, a subset of LGFMS has been reported to have a perineurioma-like morphology that frequently shows strong expression of claudin-1[17]. However, the prominent arcades of small blood vessels typical of LGFMS are usually absent in soft tissue perineurioma. Moreover, giant collagen rosette formation, a distinctive feature in a subset of LGFMS, is very uncommonly seen in soft tissue perineurioma[18]. LGFMS is genetically characterized by t(7;16)(q33;p11) with resultant *FUS-CREB3L2* fusion in approximately 75 % of cases. Rare cases harboring fusion variants of *FUS-CREB3L1* and *EWSR1-CREB3L1* have been described [19]. MUC4, an epithelial glycoprotein, has been found to serve as a highly sensitive and specific marker for LGFMS and is expressed in approximately 99 % of cases with usually diffuse and strong staining [20]. In a study by Doyle et al. [20], MUC4 was found to be negative in all forty cases of soft tissue perineurioma included and was indicated to be helpful for the differentiation between LGFMS and perineurioma. Most recently, Plus et al. [21]found that tumors within the spectrum of LGFMS and SEF but lacking MUC4 expression and *FUS/EWSR1-CREB3L* fusion harbored a novel *YAP1-KMT2A* fusion, potentially expanding the genetic spectrums of these related tumors. In case 1 of our report, MUC4 was expressed in less than 10 % tumor cells, raising the suspicion of LGFMS. However, positivity for CD34 by IHC, which is very uncommon in LGFMS, and negativity for rearrangements of *FUS* and *EWSR1* by FISH analyses excluded the possibility of LGFMS. Both our cases were negative for *KMT2A* rearrangements, which can further help with differentiation.

Cytogenetically, soft tissue perineurioma shares similar pathogenic mechanisms with those of other nerve sheath tumors. Deletion of 22q12 and mutations in *NF2* are the most frequently reported genetic abnormalities. Deletion of 17q11 (including *NF1*) is also a recurrent event in a subset of cases [5]. In the present report, we performed targeted NGS for 425 cancer-relevant genes, including *NF1* and *NF2*, in both tumors, which revealed low levels of TMB and likely pathogenic mutations with *CYP2B6* and *FLT1* mutations for 1 each; however, no genomic alterations in *NF1* or *NF2* were identified. *CYP2B6*, located at 19q13.2, is a polymorphic detoxification gene that plays a vital role in the degradation of genotoxic compounds. Single nucleotide polymorphisms in the *CYP2B6* gene locus, which result in reduced enzymatic activity, have been associated with many types of solid tumors, mainly hematological malignancies [22]. *FLT1*, also known as *vascular endothelial growth factor receptor-1 (VEGFR-1)*, is located at 13q12.3 and encodes a member of the VEGFR family. VEGFR family members are receptor tyrosine kinases and serve as critical mediators of tumor angiogenesis and vessel permeability. VEGFR-1 has been detected in schwannoma, and increased levels of this factor correlate with increased rates of tumor growth [23]. Mutations of *CYP2B6* and *FLT1* have not been reported in soft tissue perineurioma, and the pathogenic significance of these mutations is largely unknown. It is worth collecting more cases for further in-depth study to determine whether these are novel potential pathogenic mutations in soft tissue perineurioma.

In summary, soft tissue perineurioma rarely arises from the kidney and is usually located in the capsular areas. Although atypical histologic features such as local invasive growth can be seen, the clinical behavior is benign. Low-grade DDLPS and LGFMS as well as other spindle cell lesions should be considered in the differential diagnosis.

## Data Availability

Records and data pertaining to both the cases are in the patient’s secure medical records in Zhejiang Provincial People’s Hospital, People’s Hospital of Hangzhou Medical College. All searched data by literature review are included in this paper.

## Abbreviations

CT: computed tomography
DDLPS: dedifferentiated liposarcoma
EMA: epithelial membrane antigen
FISH: fluorescence in situ hybridization
GFAP: glial fibrillary acidic protein
GLUT1: glucose transporter 1
IHC: immunohistochemistry
LGFMS: low-grade fibromyxoid sarcoma
NGS: next generation sequencing
SEF: sclerosing epithelioid fibrosarcoma
SMA: smooth muscle actin
TMB: tumor mutation burden
VAF: variant allele frequency
VEGFR-1: vascular endothelial growth factor receptor − 1
